# Amino acid requirements of the infant: the amino acid composition of human breast milk

**DOI:** 10.3389/fnut.2024.1446565

**Published:** 2024-09-17

**Authors:** Paul J. Moughan, Amelie Deglaire, Yalu Yan, Philip Wescombe, Wen Xin Janice Lim, Natascha Stroebinger, Sufang Duan, Ignatius Man-Yau Szeto, Suzanne Hodgkinson

**Affiliations:** ^1^Riddet Institute, Massey University, Palmerston North, New Zealand; ^2^INRAE, Institut Agro, STLO, Rennes, France; ^3^Inner Mongolia Dairy Technology Research Institute Co. Ltd., Hohhot, China; ^4^Oceania Dairy Limited, Glenavy, New Zealand; ^5^National Center of Technology Innovation for Dairy—Oceania Innovation Center, Lincoln, New Zealand; ^6^National Center of Technology Innovation for Dairy, Hohhot, China

**Keywords:** breast milk, human milk, human milk protein, indispensable amino acid, infant nutrition, lactation, protein hydrolysis, true ileal amino acid digestibility

## Abstract

The recommended amino acid requirements of the infant are based on the amino acid composition of mature human breast milk. The amino acid composition of breast milk is usually determined following either acid or alkaline (for tryptophan) hydrolysis. For accuracy, however, the known effect of hydrolysis time on amino acid composition should be accounted for. Also, ideally the amino acid composition of breast milk should be given in units of digested (assumed to be absorbed) amino acids. A review of the literature is presented which gives mean total amino acid concentrations in mature human milk (*n* = 26 studies), mean hydrolysis correction factors (*n* = 3 studies) and mean true ileal amino acid digestibility coefficients (*n* = 3 studies, suckling piglet). There were differences between the estimates of amino acid concentration corrected for hydrolysis time and digestibility, and current FAO (2013) recommendations that were not corrected for these factors. The values based on the published literature up until 2023 (mg/g true protein) corrected for hydrolysis time and digestibility gave higher values (more than 16% higher) for leucine, lysine and threonine, and considerably higher values (greater than 30%) for histidine and tryptophan. Current recommendations may need revision.

## 1 Introduction

Human breast milk is a complex biological fluid and in nature is the sole source of nutrients for a baby for the first few months of life. The protein composition and consequent amino acid composition of breast milk is the result of millions of years of evolution, and as such, it is generally assumed that the amino acid composition of breast milk from healthy well-nourished women, provides a suitable basis for estimates of the amino acid requirements of the baby postnatally (1, 2). It is of utmost importance to know the amino acid requirements of the infant with accuracy as they provide the building blocks of proteins synthesised during growth and development, and many of the amino acids have important specific physiological roles (3–5).

Breast milk is the preferred source of nutrition for the newborn baby, but for numerous reasons in practice many infants receive infant formula as their sole source of nutrition. It is important, therefore, to have accurate estimates of the amino acid composition of breast milk.

Human milk contains hundreds of different proteins of which the concentrations are variable, and although the amino acid sequences of some of the more common milk proteins are known, not all of the proteins have been sequenced. Moreover, a significant proportion of breast milk amino acids are in the free form. It is for these reasons that the amino acid composition of milk is usually determined by chemical analysis.

Since the development of ion-exchange chromatography and other methods such as HPLC and UHPLC with precolumn derivatization to separate amino acids in complex mixtures, many studies have been reported determining the amino acid composition of human milk. Common to these studies is the need to firstly hydrolyse the breast milk proteins to their constituent free amino acids to allow quantitation. This commonly involves acid hydrolysis (usually 6 M HCl) of the defatted material in an oxygen free environment for 20 to 24 h at 110 degrees Celsius. It is well established, however, that with strong acid hydrolysis, methionine (particularly if oxygen is present), cysteine and tryptophan can be destroyed. Accordingly, methionine and cysteine are usually determined as methionine sulphone and cysteic acid, respectively following performic acid oxidation undertaken before the hydrolysis step, and tryptophan after an alkaline hydrolysis. Also, during hydrolysis tyrosine can become halogenated but this can be prevented by adding phenol to the hydrolysis mixture. What is less widely appreciated, however, is that regardless of the type of hydrolysis, a hydrolysis time longer than 24 h is required for the full release of some amino acids (for example leucine, isoleucine and valine), while others (for example serine, threonine, cysteic acid, tryptophan) can be progressively oxidized (6). The degree of underestimation can be practically important urging some authorities to adopt correction factors (e.g., TNO, the Netherlands: threonine 1.05; serine 1.10; valine 1.07; isoleucine 1.08). For some applications such a degree of underestimation may be acceptable, but it is important that infant formulas mimic the amino acid composition of human milk as accurately as possible.

An approach to determining amino acids that is more accurate than using a set hydrolysis time, is to subject the protein to multiple hydrolyses (different durations of hydrolysis) and then apply a curvilinear mathematical model to allow the prediction of the amounts of amino acids present in the protein, accounting for simultaneous rates of both amino acid release and destruction (7, 8). The Robel and Crane model has been modified (9) to allow for complex mixtures, such as breast milk, that have a free amino acid as well as a bound proteinaceous amino acid component.

Another consideration when equating milk amino acid contents with amino acid requirements for the infant is that not all proteins in human milk have a primary nutrition function, but rather some proteins (e.g., immunoglobulins; lactoferrin; transferrin; lysozyme) may have primary immunological and developmental roles. It appears that these types of proteins are only partially digested between the mouth and end of the small intestine and complete fragments of such proteins can be detected in faeces from breast-fed babies (10–12). Consequently, not all human milk amino acids are absorbed and thus a more refined estimate of amino acid requirements is given by the digestible (assumed absorbed) amino acids in human milk (13). It is the profile of absorbed rather than gross amino acids that needs to be mimicked by the digestible amino acids in infant formulas.

It is well established that the absorption of intact amino acids in humans is essentially complete by the end of the small intestine and that amino acid digestibility should be determined between the mouth and the terminal ileum using a true ileal amino acid digestibility assay (14, 15). Such a measure cannot be readily obtained using human infants, necessitating the need for animal models of digestion. The three-week-old suckled piglet, ingesting milk at an amount per unit stomach volume to mimic the human infant, has been shown to be a suitable candidate model for the three-month-old human baby, from an anatomical and physiological perspective (16–19). A study directly comparing the protein and organic matter digestion of milk in the piglet and human baby, provides empirical evidence for the suitability of the suckled piglet model (20). Other models such as the rat pup have been successfully used to study the digestion of milk proteins (21) but such models rely upon intubation of the milk, and thus exclude suckling, which may affect digestion. The suckled piglet model has been used in several studies to determine the digestibility of amino acids in breast milk.

The objective of this contribution is to review the published literature on the amino acid composition of human milk with an emphasis on the effect of amino acid losses and gains during hydrolysis, and on the absorbability of the breast milk amino acids. A profile of absorbed amino acids in human milk is put forward as the current best estimate of the amino acid needs of the newborn term infant. This amino acid profile is compared with the current FAO recommendations (2). The latter recommended amino acid profile does not consider the effects of hydrolysis time during amino acid analysis, nor the effects of differences in amino acid digestibility.

## 2 Methods

A systematic review of the literature was conducted to identify publications that reported total amino acid concentrations in human milk, including publications up until the end of 2023. A search was performed using Scopus and Google Scholar. Keywords used were “PubMed”, “amino acid,” “protein composition,” “human milk composition,” “human milk,” “breast milk,” “human milk nutrition,” “characterisation of human milk,” “standardisation of human milk,” and “factors affecting human milk composition.” Reference lists of the selected publications were further searched manually to identify any other relevant articles. A total of 74 articles were identified for potential inclusion.

Within each publication, milk collection methods and methods for amino acid analysis were reviewed. Inclusion criteria included the collection of mature milk from healthy women, defined as collection periods extending between beyond 1 to 10 months post-partum. For publications that presented total amino acid concentrations for multiple lactation periods, those that were within the specified collection period were averaged.

Exclusion criteria included collection from a single donor or from non-healthy women or results from the collection of non-mature milk (defined as less than 1 month post-partum). Studies that conducted amino acid analysis using non-standard methods were also excluded. Publications in which the milk collection methodology or amino acid analysis methods were not well-described were excluded.

When the results from one study were reported in several publications (determined according to the description of methods), these data were only included in the database once. Table 1 lists the studies that were not included in the database and the reason for their exclusion.

**Table 1:** Studies that report the amino acid (AA) concentration in mature human milk that were published before 2023 and not included in the present dataset.

Study	Exclusion criteria
Atkinson et al. (52)	Data from collection periods earlier than 1 month post-partum
Beach et al. (53)	Non-standard amino acid analysis (microbiological)
Block and Bolling (54)	Non-standard amino acid analysis (microbiological)
Chathyushya et al. (55)	Only breast milk of 1 week post-partum collected
Close and Van De Walle (56)	Inadequate information on breast milk sample
Darling et al. (57) and Darling (58)	Data from collection periods earlier than 1 month post-partum
Davis et al. (59)	Data reported in another publication that was included (duplicate data)
DeSantiago et al. (60)	Health criteria of lactating mothers not met
Faus et al. (61)	Total AAs not determined
Feng et al. (62)	Data reported in another publication that was included (duplicate data)
Ferreira (63)	Data from collection periods earlier than 1 month post-partum
Filippova and Aronova (64)	Method for AA determination not reported
Giuffrida et al. (65)	Total AAs not determined
Guo et al. (66)	Single milk donor
Hanning et al. (67)	Data from collection periods earlier than 1 month post-partum
Heine et al. (51)	Data reported in another publication that was included (duplicate data)
Jarvenpaa et al. (68)	Data reported in another publication that was included (duplicate data)
Lemons et al. (69)	Total AAs not determined
Macy (70) and Macy and Kelly (71)	Non-standard amino acid analysis (microbiological)
Miller et al. (72)	Non-standard amino acid analysis (microbiological)
Mitton and Garlick (73)	Inadequate information on breast milk sample
Moya-Alvarez et al. (74)	Health criteria of lactating mothers not met (many with malnutrition)
Motil et al. (75)	Total AAs not determined
Nagasawa et al. (76)	AA profile of breast milk casein only
Nayman et al. (77)	Non-standard amino acid analysis (microbiological)
Nwachoko et al. (78)	Results reported in mg AA/100 g protein but no total N or protein data provided
Pang et al., 2019 (79)	Inadequate information on methodology; text in Mandarin
Picone et al. (80)	Inadequate information on methodology
Purkiewicz et al. (81)	Data from collection periods earlier than 1 month post-partum
Räihä et al. (82)	Data reported in another publication that was included (duplicate data)
Rassin et al. (83)	Lactation stage unclear
Renner (84)	Data reported in another publication that was included (duplicate data)
Rigo et al. (85)	Data reported in another publication that was included (duplicate data)
Saben et al. (86)	Total AAs not determined
Saito et al. (87)	Non-standard amino acid analysis (microbiological)
Sarwar et al. (88)	Data from collection periods earlier than 1 month post-partum
Scott et al. (89)	Inadequate information on methodology
Shaikhiev (90)	Text in Russian (Cyrillic)
Soupart et al. (91)	Lactation stage unclear
Tarján et al. (92)	Total AAs not determined
Tikanoja et al. (93)	Lactation stage unclear
Van Sadelhoff et al. (94)	Total AAs not determined
Volz et al. (95)	Data reported in another publication that was included (duplicate data)
Wei et al. (96)	Total AAs not determined; non-standard amino acid analysis; not representative of mature milk
Williamson (97)	Non-standard amino acid analysis (colorimetric)
Woodward (98)	AA profile of breast milk casein only

A total of 26 studies were included in the database (13, 22–46).

The amino acid composition of human milk was reported using different units in the publications so these were converted when required, to mg amino acid/L milk, mg amino acid/g dry matter (DM) and mg/g true protein (TP). Data that were presented only in moles were first converted to mg of amino acid per L milk by multiplying by the molecular weight of each amino acid (x 10). For each publication, the reported dry matter (DM) content of milk given in that publication was used to convert each amino acid value between mg/L and mg/g DM. When the DM content of the milk was not reported in the publication, the average DM content of milk calculated from publications that reported this value was used (121.2 mg DM/L milk). To convert between mg/g and mg/L, the conversion factor of 1.032 mg milk/L was used (47).

Values were also converted to mg/g true protein (TP) with the TP content of the milk samples calculated as reported by FAO (2) where TP = nitrogen concentration × 6.38 × 0.75. The factor of 0.75 is used as the non-protein content of human milk (comprising mainly urea and free amino acids) is around 25% of the total nitrogen content (2). Where necessary, as different publications used different conversion factors between nitrogen concentration (which is chemically analysed) and crude protein, reported protein concentrations were first converted back to nitrogen concentrations according to the reported conversion factor in each publication.

A review of the literature (until 2023) was also undertaken to identify studies addressing the effect of hydrolysis time on amino acid yield in human breast milk, and studies determining the true ileal digestibility of amino acids in human breast milk.

## 3 Results and discussion

### 3.1 Amino acid composition

The overall mean total amino acid compositions of human milk reported in the 26 studies included in the database are given in Table 2. Zhang et al. (48) also conducted a systematic review of the total amino acid concentration in human milk, and their values are included in Table 2.

**Table 2:** Amino acid composition of human milk (based on 20 to 24 h amino acid hydrolysis period) collected from women between 3 and 42 weeks post-partum from data published before 2023 (*n* = 26 studies) and values reported in the systematic review of Zhang et al. (48).^1^

	Amino acid composition			
Amino acid	mg/L	mg/g DM^2^	mg/g TP^3^	Zhang et al. (48) mg/L^1^
Indispensable amino acid				
Histidine	280.8 ± 12.65	2.3 ± 0.11	31.2 ± 1.47	278.0
Isoleucine	572.7 ± 15.68	4.7 ± 0.15	63.6 ± 1.81	597.5
Leucine	1084.4 ± 29.47	9.0 ± 0.26	120.0 ± 3.10	1117.0
Lysine	755.0 ± 23.85	6.2 ± 0.20	83.8 ± 2.73	755.5
Methionine	162.2 ± 6.60	1.3 ± 0.06	18.0 ± 0.71	172.0
Phenylalanine	420.8 ± 16.60	3.5 ± 0.14	46.8 ± 2.00	425.0
Threonine	499.0 ± 14.22	4.1 ± 0.13	55.2 ± 1.48	510.5
Tryptophan^4^	196.1 ± 11.84	1.5 ± 0.18	21.6 ± 1.30	222.0
Valine	596.7 ± 19.31	4.9 ± 0.19	66.3 ± 2.18	625.0
Dispensable amino acid				
Alanine	422.1 ± 16.36	3.5 ± 0.15	46.9 ± 1.72	436.5
Arginine	411.2 ± 19.80	3.4 ± 0.17	45.6 ± 2.14	409.5
Aspartic acid	971.5 ± 26.13	8.0 ± 0.23	107.8 ± 2.88	990.5
Cysteine^5^	233.2 ± 863	1.8 ± 0.15	26.3 ± 0.98	237.0
Glutamic acid	1898.2 ± 37.87	15.7 ± 0.38	211.1 ± 4.96	1952.5
Glycine	253.8 ± 12.59	2.1 ± 0.11	28.1 ± 1.38	266.0
Proline	937.1 ± 26.35	7.8 ± 0.26	103.9 ± 3.02	976.0
Serine	492.2 ± 14.29	4.1 ± 0.13	54.4 ± 1.23	499.5
Tyrosine	456.6 ± 24.97	3.8 ± 0.21	51.0 ± 2.96	515.0

Values are given as mean ± SEM between studies.

1 Data averaged from milk collected between 3 and 20 weeks postpartum, Zhang et al. (48).

2 DM, dry matter; when no dry matter content of breast milk was given in the original publication an average of the collected data (*n* = 6) was used.

3 TP, true protein = N × 6.38 × 0.75.

4 Tryptophan values from published studies were only included when an alkaline hydrolysis was performed (*n* = 12).

5 Cysteine values from published studies were only included when an initial treatment of the sample with performic acid was applied (*n* = 15).

The rigorous and detailed review of Zhang et al. (48) covering the literature published up to 2009 provides an important benchmark against which to compare the presently derived data. Data included in the Zhang et al. (48) study related to breast milk samples from complete 24 h collections or at least collections of the entire amount of milk from one or both breasts at a feeding, or pooled or banked milk. The milk was from healthy mothers receiving “free-living” diets and who had delivered healthy mainly term babies. Studies employing microbiological methods of amino acid determination were excluded, and only studies using ion exchange chromatography, HPLC and UHPLC with precolumn derivatization or similar validated methods were used in the analysis. Results largely relate to 20 to 24 h hydrolysis of protein. Attention was paid to ensuring that the studies used appropriate consistent methods for the determination of methionine, cysteine and tryptophan. Overall mean amino acid concentrations in milk were determined for each study and least squares means generated with stage of lactation fitted as an effect in the ANOVA model. Mature milk in the Zhang et al. (48) work was defined as milk from 21 days of lactation up to >136 days of lactation. Lactation stage significantly (*p* < 0.05) influenced total amino acid composition and data were presented separately for mature milk relating to 21 to 58 days of lactation; 59 to 135 days of lactation and 136 to 540 days of lactation. Most studies used the units of weight of amino acid per 100 ml milk. Where data were given as weight per 100 grams milk, the volume-weight correction, which is quantitatively minor, was not undertaken. The study reviewed human milk composition data from 83 published scientific papers, from 18 countries, with publication dates ranging from 1941 to 2009. For total amino acid content, 26 papers providing 79 mean values from 3,774 subjects were selected by the authors for analysis. The total N concentration of breast milk and the amino acid content of breast milk declined (*p* < 0.05) moderately for milk from around 2 months of lactation to milk from 5 to 18 months of lactation. This is consistent with the conclusions reached by Lönnerdal et al. (49) and Ren et al. (47) that human milk amino acid content is relatively stable from around 3 to 4 weeks after birth and onwards. For our purposes and to align with the lactation period used in the present work, the mean concentrations calculated over 21 to 135 days of lactation were taken as an estimate of the amino acid composition of mature human breast milk (see Table 2).

There is close agreement between the values for the amino acid composition of human breast milk between Zhang et al. (48) and the present estimates, though differences were found for some of the amino acids. This gives confidence in the presently derived estimates. The presently reported estimates are preferred, as these incorporate the most up-to-date published information (studies published up to 2023, as opposed to 2009).

### 3.2 Correction for the effect of time of hydrolysis

Three studies were identified that conducted amino acid analysis with multiple hydrolysis intervals (13, 44, 45). The difference between the concentration of each amino acid determined using a 20 to 24 h hydrolysis period and that determined using multiple hydrolyses (estimated amino acid concentration in the milk) for each amino acid in each study was averaged to calculate average correction factors. These correction factors were used to correct the total amino acid concentration in human milk (reported in Table 2; based on 20 to 24 h hydrolysis) to that if multiple hydrolysis had been used for each individual published study, and the mean results are reported in Table 3. In the publication by Charton et al. (44) data corresponding to 24 h hydrolysis were not included, but data were provided (A. Deglaire, personal communication) to allow the correction to be made.

**Table 3:** Determined correction factors^1^ for breakdown or incomplete release of amino acids during hydrolysis, and concentration of amino acids in breast milk corrected by these values.

Amino acid	Correction factor (%)	Value before correction mg/L (20 to 24 h hydrolysis)^2^	Value after correction		
			mg/L	mg/g DM^3^	mg/g TP^4^
Indispensable amino acid					
Histidine	4.7	280.8	294.0	2.4	32.7
Isoleucine	−0.3	572.7	571.0	4.7	63.4
Leucine	−1.3	1084.4	1070.3	8.8	118.7
Lysine	−0.7	755.0	749.7	6.2	83.2
Methionine	−0.7	162.2	161.0	1.3	17.8
Phenylalanine	−2.0	420.8	412.4	3.4	45.9
Threonine	6.2	499.0	529.9	4.4	58.8
Tryptophan	2.0	196.1	200.0	1.5	22.0
Valine	1.5	596.7	605.6	5.0	67.2
Dispensable amino acid					
Alanine	−1.9	422.1	414.0	3.4	46.0
Arginine	−1.4	411.2	405.4	3.4	45.0
Aspartic acid	0.2	971.5	973.4	8.0	108.0
Cysteine	−3.7	233.2	224.5	1.8	25.3
Glutamic acid	−0.4	1898.2	1890.6	15.6	210.2
Glycine	−2.7	253.8	247.0	2.0	27.3
Proline	0.5	937.1	941.8	7.8	104.5
Serine	5.0	492.2	516.8	4.3	57.1
Tyrosine	3.2	456.6	471.2	3.9	52.6

1 Correction factors are mean differences between concentration of amino acids determined with 24 h hydrolysis and multiple hydrolysis intervals (modelled value), based on observations from Darragh and Moughan (13), Charton et al. (44) and Hodgkinson et al. (45). Values using the equation below for each amino acid in each of the three studies expressed as a percentage were averaged: Correction value = (Modelled concentration of amino acid) − (Concentration of amino acid using 20–24 h hydrolysis)/Concentration of amino acid using 20–24 h hydrolysis.

2 Data from Table 2.

3 DM, dry matter; when no dry matter content of breast milk was given in the original publication an average of the collected data (*n* = 6) was used.

4 TP, true protein = N × 6.38 × 0.75.

For most of the amino acids the effect of correction was small but for histidine, phenylalanine, threonine, tryptophan, cysteine, serine and tyrosine the differences were considered to be practically important (correction factor ≥ 2%). For leucine, isoleucine and valine the determined correction factors were smaller than expected, though the hydrolysis behaviour is likely to vary with the substrate being analysed. The amino acid affected by hydrolysis to the greatest extent was threonine, which is known to be sensitive to oxidation (6).

### 3.3 Correction for the true ileal amino acid digestibility

True ileal amino acid digestibility coefficients for human milk determined using the piglet as a model for the human infant were reported in three studies (13, 44, 45). For each amino acid, digestibility coefficients were averaged across the three studies (Table 4) and applied to the data presented in Table 3. The overall mean true ileal amino acid digestibility coefficients and mean amounts of true ileal digestible amino acids are presented in Table 5.

**Table 4:** Published mean true ileal amino acid digestibility of amino acids in breast milk determined using the piglet as a model for the human infant.

Amino acid	Study		
	Charton et al. (44)	Darragh and Moughan (13)	Hodgkinson et al. (45)
Indispensable amino acid			
Histidine	0.979	0.950	0.952
Isoleucine	0.963	0.980	0.955
Leucine	0.982	0.990	0.942
Lysine	0.984	0.980	0.920
Methionine	ND^1^	1.000	0.956
Phenylalanine	0.963	0.930	0.879
Threonine	0.892	0.860	0.842
Tryptophan	0.955	ND^1^	1.000
Valine	0.931	0.900	0.883
Dispensable amino acid			
Alanine	0.931	0.950	0.812
Arginine	0.965	1.010	0.842
Aspartic acid	0.949	0.950	0.905
Cysteine	ND^1^	ND^1^	0.678
Glutamic acid	0.976	0.980	0.945
Proline	0.941	0.920	0.876
Serine	0.938	0.950	0.774
Tyrosine	0.959	1.000	-

1 ND, not determined.

**Table 5:** True ileal amino acid digestibility coefficients (TIAAD)^1^ and amounts of true ileal digestible amino acids in human milk presented in different units.

Amino acid	TIAAD	Amount of digestible amino acids^2^		
		mg/L	mg/g DM^3^	mg/g TP^4^
Indispensable amino acid				
Histidine	0.960	282.3	2.3	31.4
Isoleucine	0.966	551.5	4.6	61.3
Leucine	0.971	1039.3	8.6	115.2
Lysine	0.961	720.5	6.0	80.0
Methionine	0.978	157.5	1.3	17.5
Phenylalanine	0.924	381.1	3.1	42.4
Threonine	0.865	458.4	3.8	50.8
Tryptophan	0.978	195.6	1.5	21.6
Valine	0.905	548.1	4.5	60.9
Dispensable amino acid				
Alanine	0.898	371.8	3.1	41.3
Arginine	0.939	380.7	3.1	42.2
Aspartic acid	0.935	910.2	7.5	101.0
Cysteine	0.678	152.2	1.2	17.2
Glutamic acid	0.967	1828.2	15.1	203.3
Glycine	0.924	228.2	1.9	25.2
Proline	0.912	858.9	7.1	95.3
Serine	0.887	458.4	3.8	50.7
Tyrosine	0.980	403.8	3.3	45.1

1 Overall mean values from Table 4.

2 Correction of values in Table 3 (after correction for time of hydrolysis effect) for TIAAD.

3 DM, dry matter; when no dry matter content of breast milk was given in the original publication an average of the collected data (*n* = 6) was used.

4 TP, true protein = N × 6.38 × 0.75.

For most of the amino acids, digestibility was high, but for some amino acids, notably cysteine and threonine, true ileal amino acid digestibility was much lower than for the other amino acids. The digestibility of threonine was consistently lower across the three studies, but only one of the studies provided digestibility data for cysteine. Mavromichalis et al. (50) reported high amino acid digestibility for sow’s milk (95–100%), but also found relatively low true ileal digestibility for cysteine and threonine (84%). Although the suckling piglet is a well-accepted animal model for protein digestion in the human infant, there may be differences in digestion (e.g., differences in gut microbial populations), and this should be borne in mind in interpreting the results.

### 3.4 Comparison with FAO recommendations

The amounts of true ileal digestible amino acids determined from the literature, corrected for multiple hydrolysis time and true ileal digestibility are presented in Table 6 along with the current FAO reference values. The FAO (2) Expert Consultation recommended the amino acid content of breast milk as the current best estimate of amino acid requirements for infants, and gave a recommended amino acid profile for mature human milk based on the deliberations of the FAO/WHO/UNU (1) Expert Consultation. FAO (2) accepted the appropriateness of correcting the total amino acid contents for amino acid digestibility, but did not make the correction at that time as only one published set of values for digestibility was available. The FAO (2) recommended values also relate to only a few older studies on the amino acid composition of human milk (37, 38, 51).

**Table 6:** Absorbed amino acid composition of human milk based on the present work (Table 5) compared with reference values from FAO (2).^1^

Amino acid	Calculated values^3^	FAO
Histidine	31	21
Isoleucine	61	55
Leucine	115	96
Lysine	80	69
Methionine + cysteine	35	33
Phenylalanine + tyrosine	87	94
Threonine	51	44
Tryptophan	22	17
Valine	61	55

Amino acid values are mg/g TP^2^, as calculated by FAO (2).

1 FAO (2) recommendations are not corrected for true ileal amino acid digestibility.

2 TP = N × 6.38 ×0.75.

3 Values based on published literature with correction for the effects of hydrolysis time and true ileal amino acid digestibility.

It is apparent from the values listed in Table 6 that the estimates of amino acid requirements for the human baby as determined after correcting published values for the amino acid content of mature human milk for the effects of hydrolysis time and true ileal amino acid digestibility, are quite different from the most recent FAO recommendations (2). The values presented here and based on the published literature up until 2023, and corrected for the estimated effect of hydrolysis time during amino acid analysis and for true ileal amino acid digestibility, led to higher concentrations (more than 16%) in breast milk for leucine, lysine and threonine and considerably higher values (greater than 30%) for histidine and tryptophan. All of these amino acids play critical roles in infant growth and development (5). A potential limitation of the present data is that although they are based on multiple studies, the hydrolysis correction factors and amino acid digestibility estimates are based on three studies only, and more investigation of these important aspects is required. In addition to the effects of the correction for hydrolysis time and amino acid digestibility, potential differences due to factors such as population (ethnic and nutritional differences), methodology related to milk collection, and advances in amino acid analysis are all undoubtedly important. Nevertheless, the corrected values shown in Table 6 are put forward as the currently most accurate estimates for the absorbed amino acid composition of human breast milk. It would seem appropriate to reassess the FAO international recommendations.
